# Supplementary material for: Structure-controllable growth of nitrogenated graphene quantum dots via solvent catalysis for selective C-N bond activation
Source: Nat Commun. 2021 Oct 7;12:5879. doi: 10.1038/s41467-021-26122-0 (PMC8497556; doi:10.1038/s41467-021-26122-0)
Supplement: Supplementary file 1 — Supplementary Information [file 41467_2021_26122_MOESM1_ESM.pdf]

Supplementary Information

Structure-controllable growth of nitrogenated graphene quantum dots via solvent catalysis for selective C-N bond activation

Byung Joon Moon,[1,2†] Sang Jin Kim,[1†] Aram Lee,[1†] Yelin Oh,[1] Seoung-Ki Lee,[3] Sang Hyun Lee,[4] Tae-Wook Kim,[5] Byung Hee Hong[2,6] and Sukang Bae[1*]

Supplementary Figures

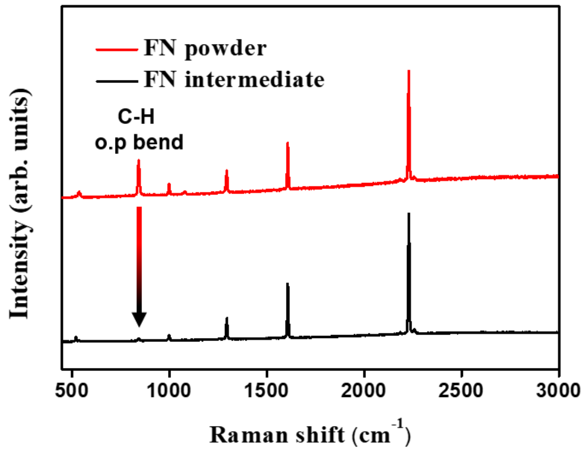

Supplementary Figure 1 Raman spectra of fumaronitrile powders and intermediate products on Si/SiO_2 substrate.

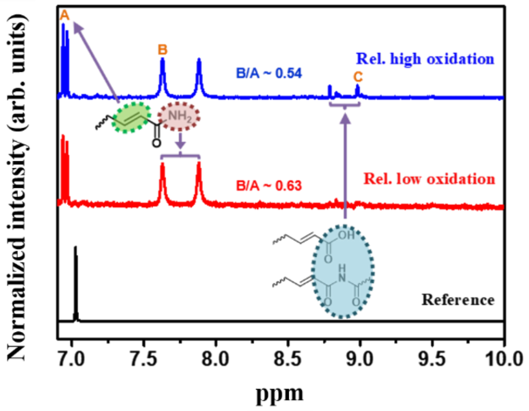

Supplementary Figure 2 ^1H NMR spectra of FN and intermediate products. These products are obtained by solvothermal oxidation at 150 °C. The oxidation degree of FN is controlled by varying the reaction time. ^1H NMR peaks are identified by following the methods in previous studies.[1,2]

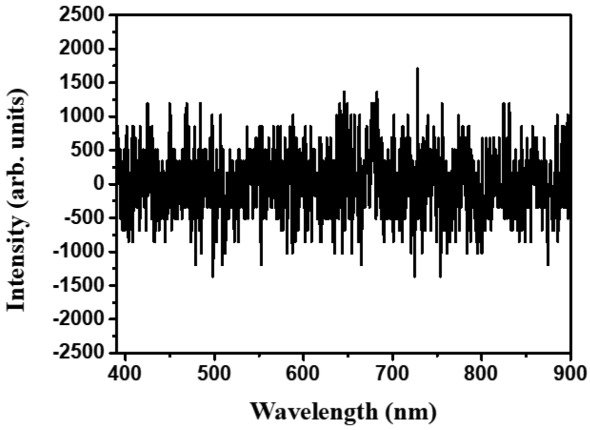

Supplementary Figure 3 PL spectrum of the intermediate product with relatively high oxidation state of FN (λ_{ex}: 375 nm).

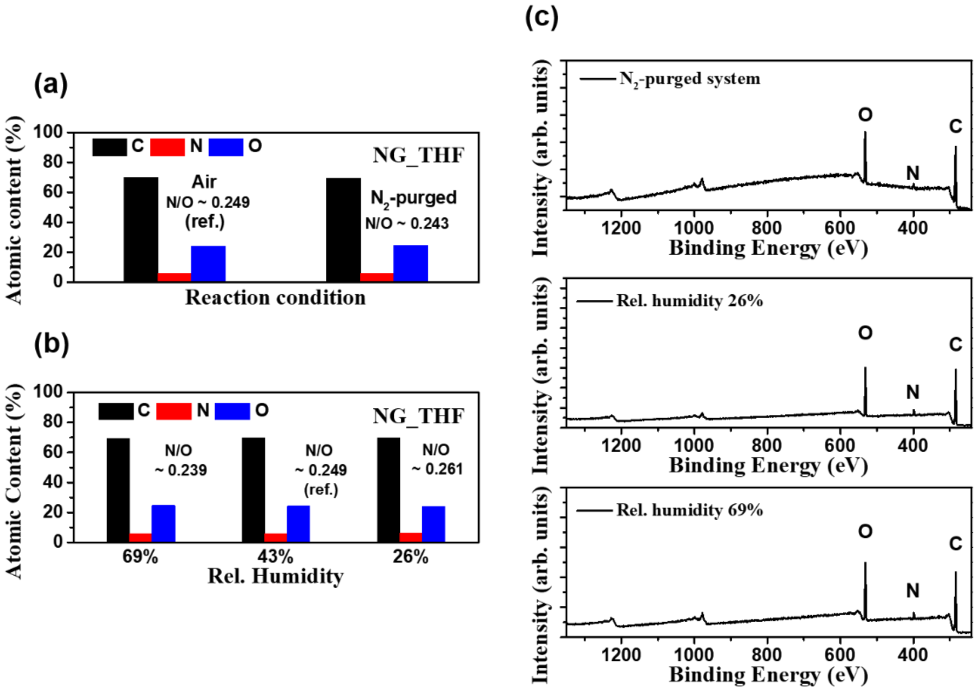

Supplementary Figure 4 **(a)** Chemical compositions of NG_THFs prepared under ambient (left) and N_2-degassed systems (right). **(b)** Chemical compositions of NG_THFs prepared under different relative humidity air conditions (26~69%). **(c)** XPS survey spectra of NG_THFs synthesized under different reaction systems (top: N_2-degassed system, middle: RH ~ 26%, bottom: RH ~ 69%).

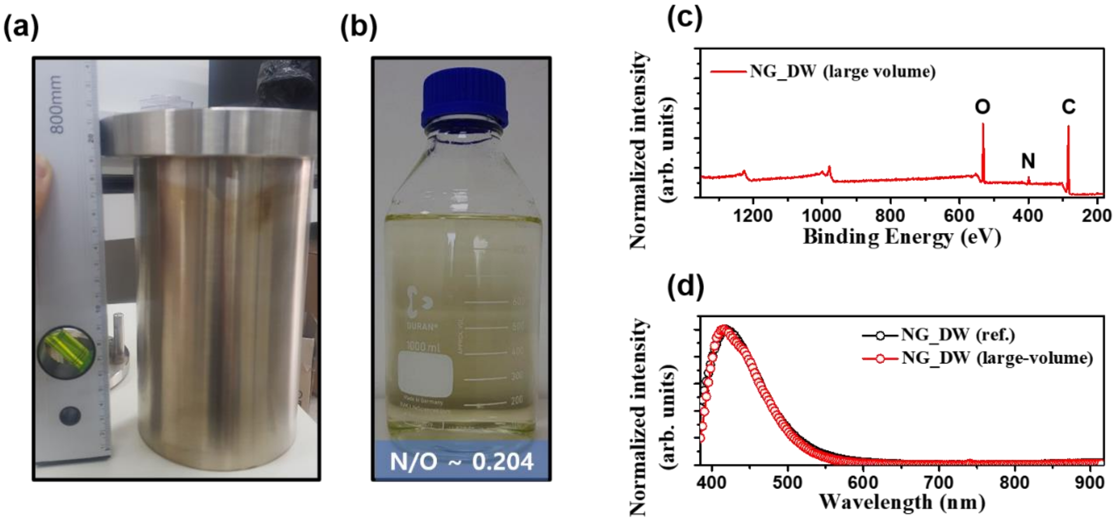

Supplementary Figure 5 (a) Photograph of apparatus for large-volume preparation of NGs. **(b)** Photograph of NG_DW dissolved in ethanol. **(c)** XPS survey and **(d)** PL spectra of NG_DW.

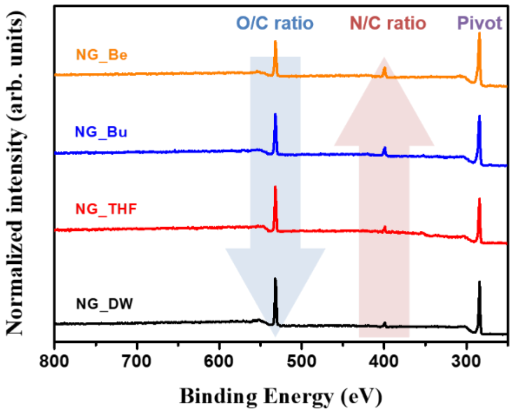

Supplementary Figure 6 Normalized XPS survey spectra of NGs synthesized under different solvent conditions. There are three major peaks at approximately 284, 400, and 533 eV, corresponding to C 1*s*, N 1*s*, and O 1*s*, respectively.

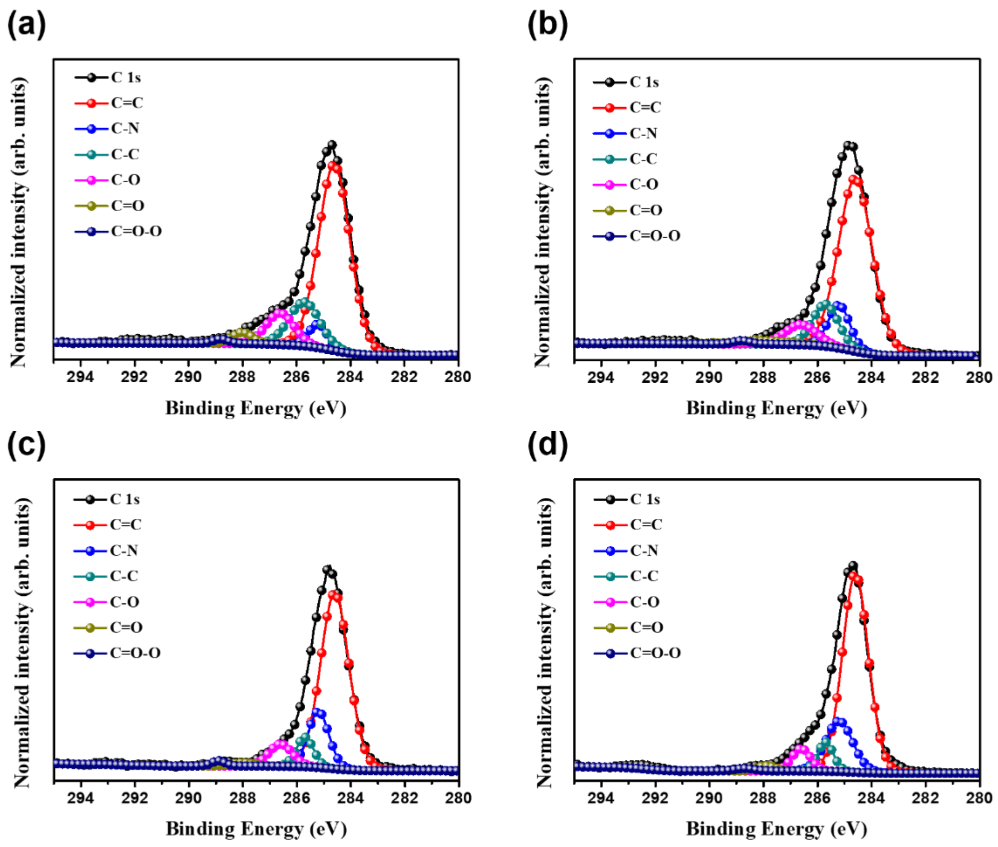

Supplementary Figure 7 C 1*s* high-resolution XPS spectra of **(a)** NG_DW, **(b)** NG_THF, **(c)** NG_Bu, and **(d)** NG_Be. These spectra can be deconvoluted into six components: the sp^2 C-C bond (C_1), C-N bond (C_2), sp^3 C-C bond (C_3), C-O bond (C_4), C=O bond (C_5), and O-C=O bond (C_6), which locate at 284.6, 285.2, 285.7, 286.6, 288.0, and 288.8 eV, respectively, in agreement with previously reported assignments.[3,4,5]

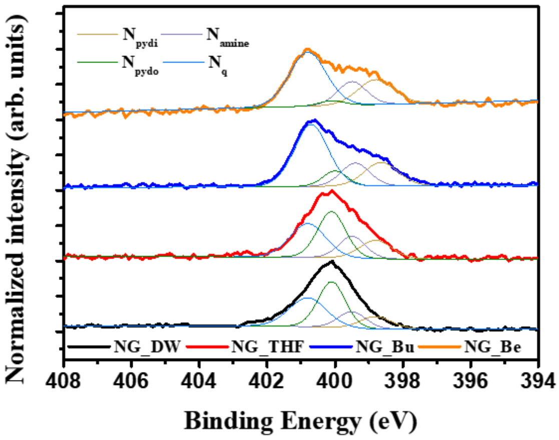

Supplementary Figure 8 N 1*s* high-resolution XPS spectra of NGs. These spectra can be deconvoluted into four signals for N_{pydi}, N_{am}, N_{pydo}, and N_q, appearing at 398.8, 399.5, 400.1, and 400.8 eV, respectively.

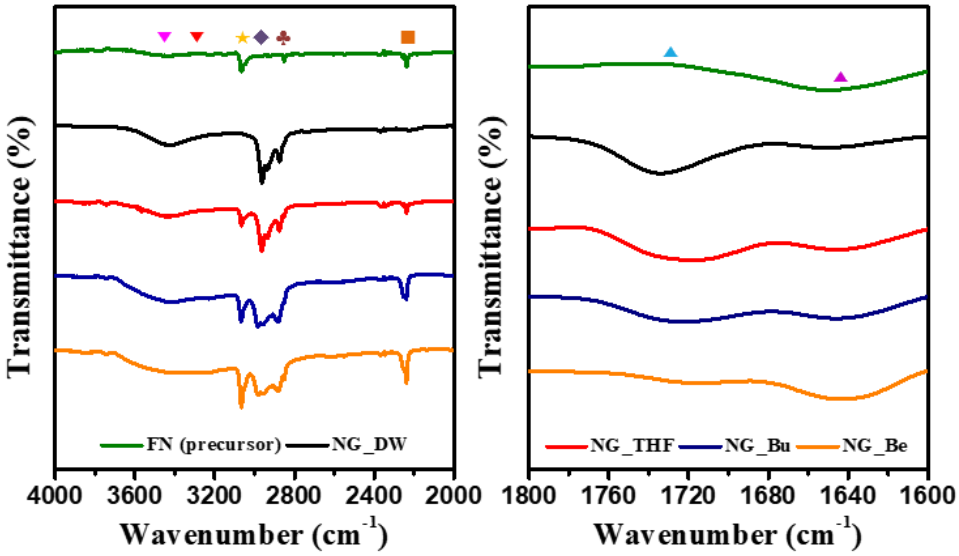

Supplementary Figure 9 FT-IR spectra of FN (precursor) and four different types of NGs. The spectrum of NG_DW is featureless except for the O-H and C=O (COOH) peaks at ~3440 (broad) and 1733 cm^{-1}, respectively, and a sharper aromatic C-H peak (bifurcated shape) at ~2965 cm^{-1}.[6,7] In addition, as the oxidation degree of FN increased (NG_Be → NG_DW), the intensities of absorption bands at ~3420 (broad) and 2240 cm^{-1}, corresponding to N-H and C≡N stretching vibrations, respectively, decrease dramatically.[8,9]

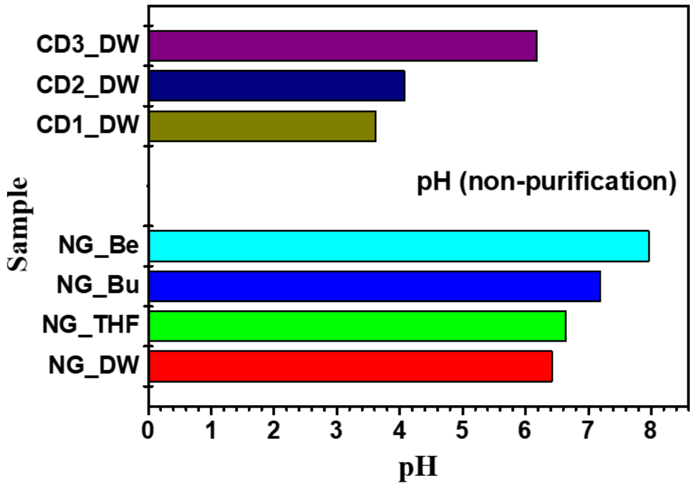

Supplementary Figure 10 pH values of CQDs as-prepared by thermolysis from various precursors. (CD1_DW: citric acid, CD2_DW: glutamic acid, and CD3_DW: glucose)

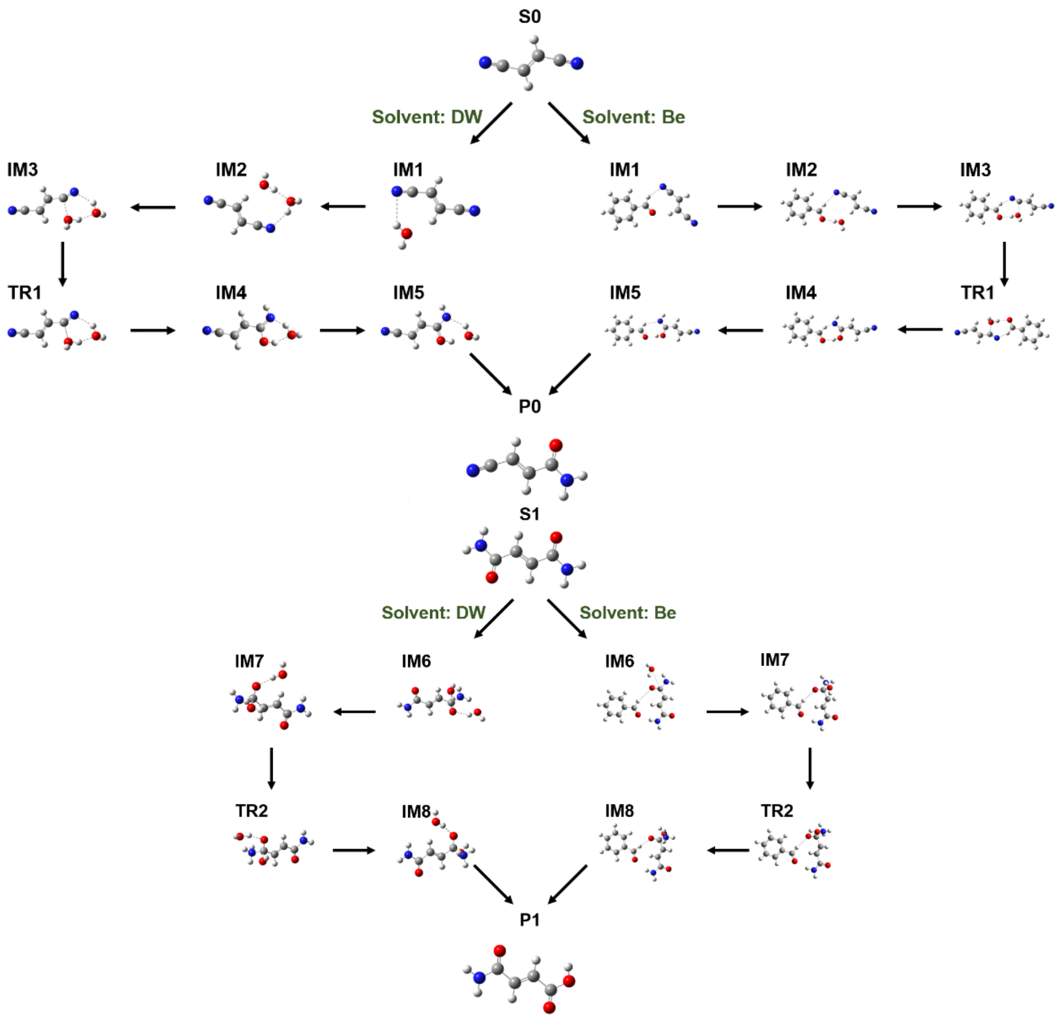

Supplementary Figure 11 Optimized structures of the reactants, intermediates, products, and transition states at each reaction pathway. The geometries of the reactant complexes, intermediates, transition states, and products of the thermolysis of FN were optimized at the B3LYP/6-311G+(d) level. These calculations were performed to compute the vibrational frequency by, 1) confirming either the obtained structures are those of transition states or local minima points and 2) estimating the zero-point vibrational energy (ZPVE).

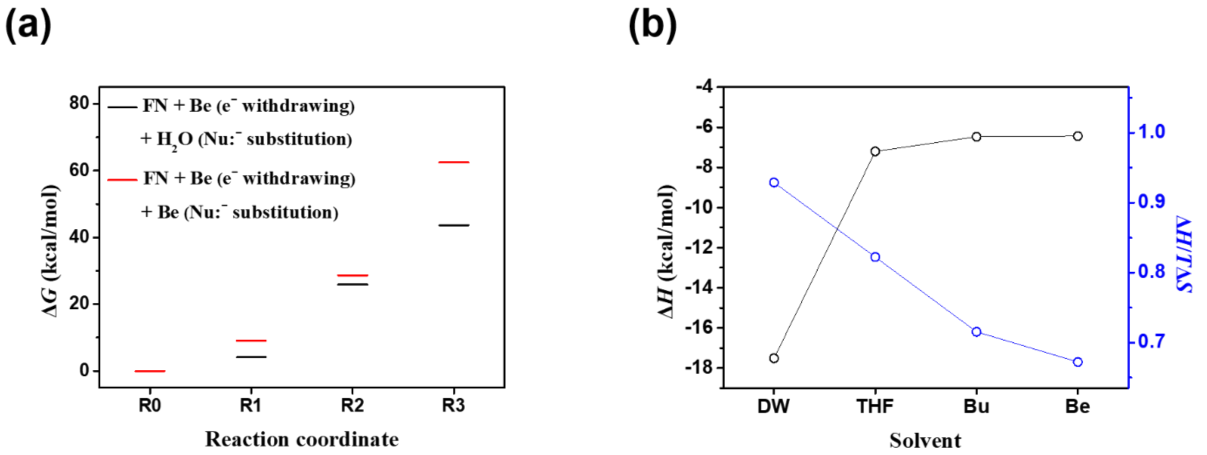

Supplementary Figure 12 (a) Free energy profiles for different reaction pathways of FN (solvent: Be) calculated at the B3LYP/6-311+G(d) level. **(b)** Thermal dynamic parameters (ΔH and $\Delta H/T\Delta S$) for the formation of IM2 complex under different solvent conditions.

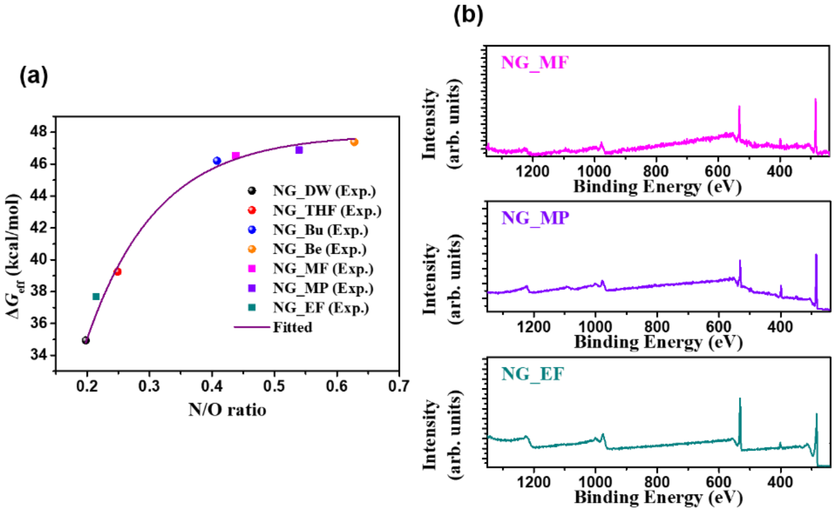

Supplementary Figure 13 (a) Applicability test of our proposed model (depicted in Figure 1g) for use as a predictable synthetic tool. **(b)** XPS survey spectra of NGs (NG_MF: *N*,*N*-Dimethylformamide, NG_EF: Ethyl formate, and NG_MP: *N*-Methyl-2-pyrrolidone).

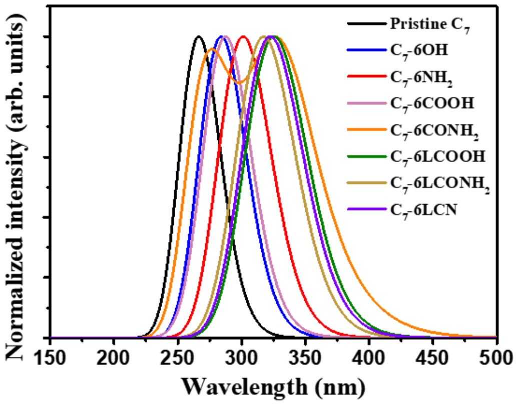

Supplementary Figure 14 Calculated absorption spectra of pristine and edge-functionalized C_7-GQDs.

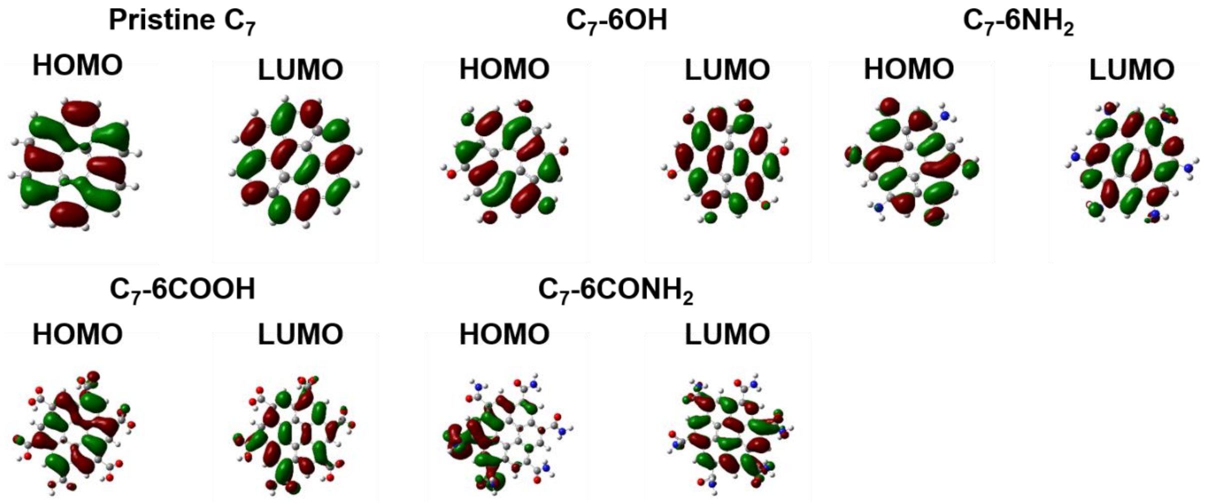

Supplementary Figure 15 Frontier molecular orbitals of various C_7-GQDs (w/o self-passivated π-linker) at the optimized geometries.

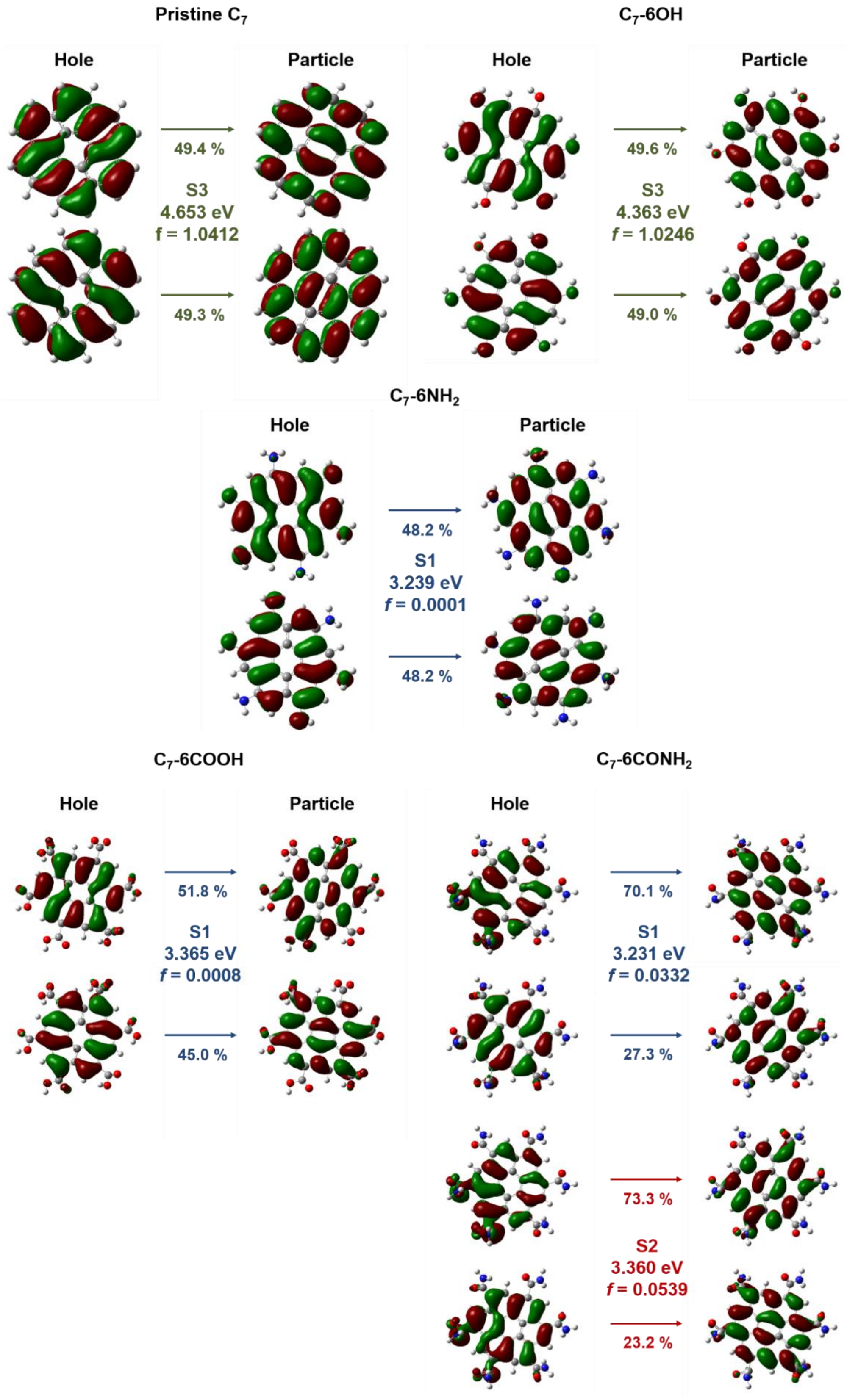

Supplementary Figure 16 NTO pairs of possible excited states of various C_7-GQDs (w/o self-passivated π-linker) with their associated eigenvalues (λ_i).

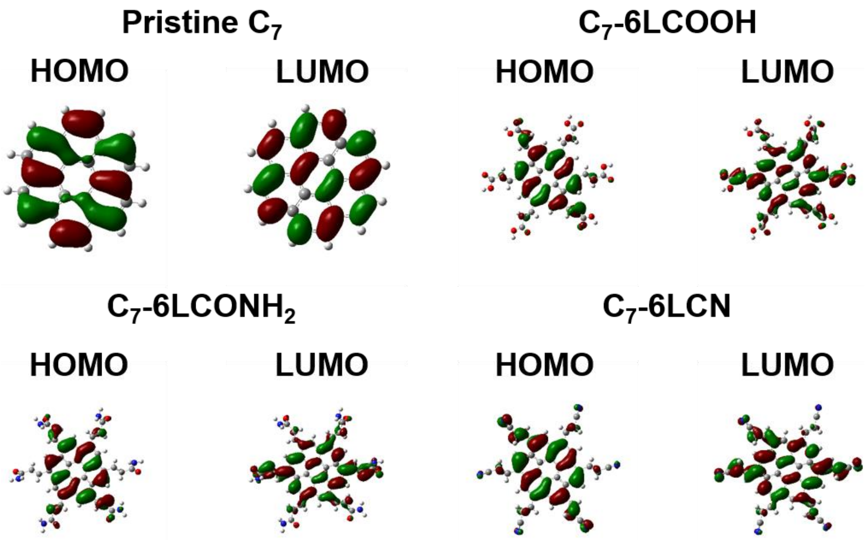

Supplementary Figure 17 Frontier molecular orbitals of various C_7-GQDs (w/ self-passivated π-linker) at the optimized geometries.

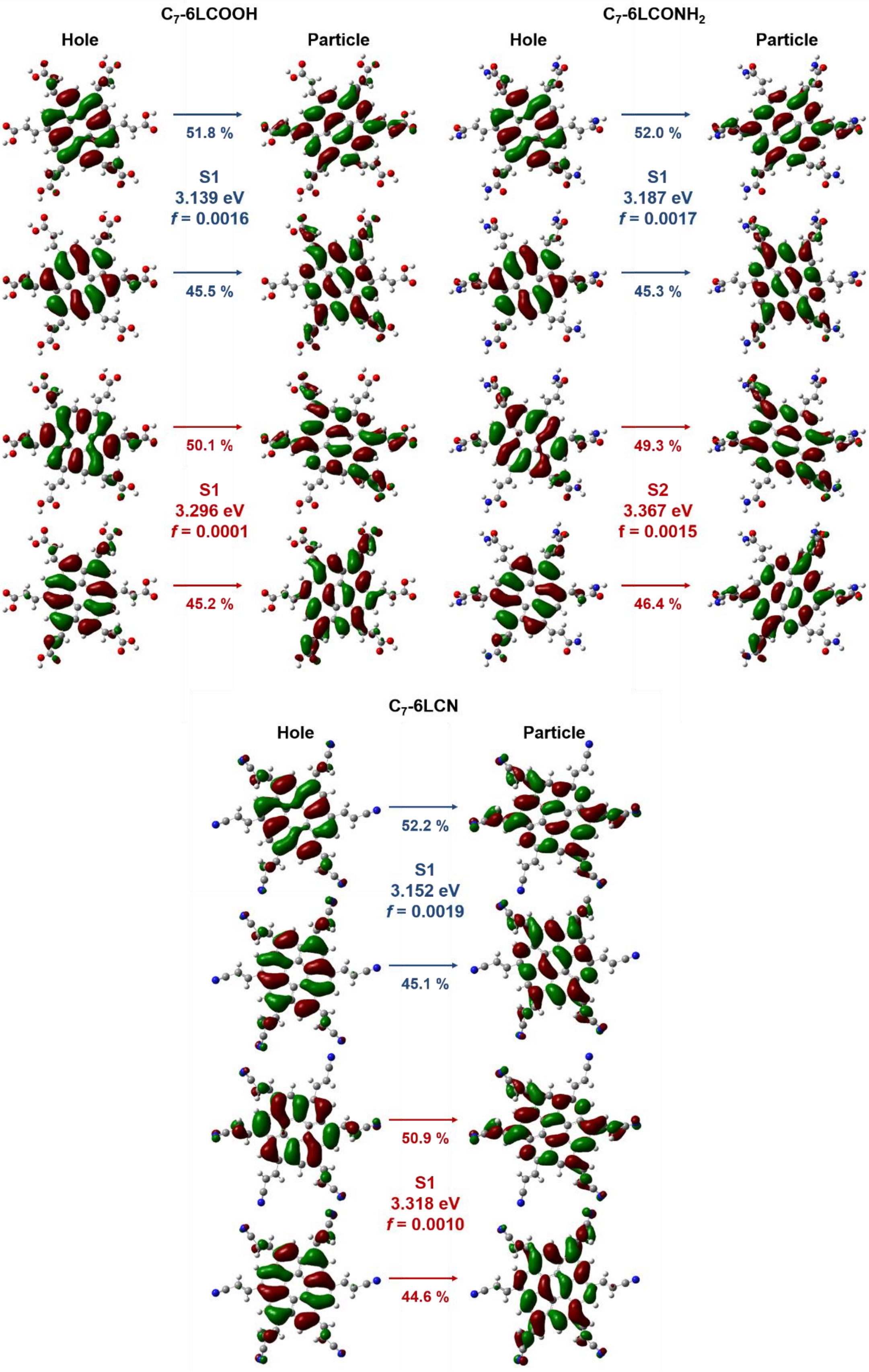

Supplementary Figure 18 NTO pairs of possible excited state of various C_7-GQDs (w/ self-passivated π-linker) with their associated eigenvalues (λ_i).

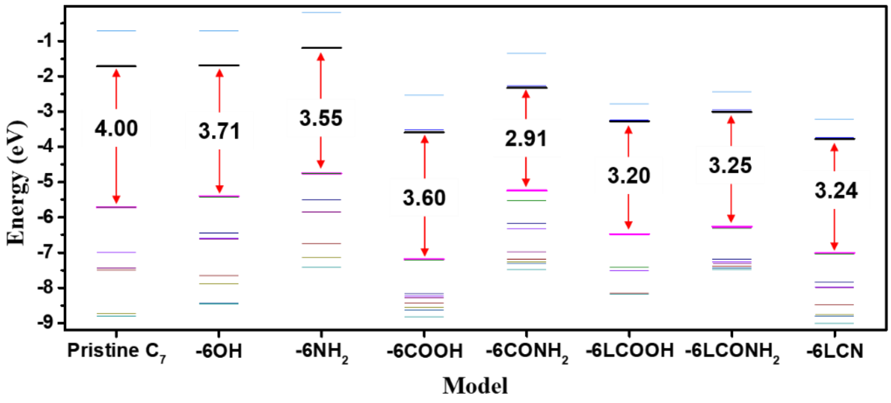

Supplementary Figure 19 Energy diagrams of C_7-GQDs with different edge-functionalization groups. (HOMO – 8 ~ LUMO + 2)

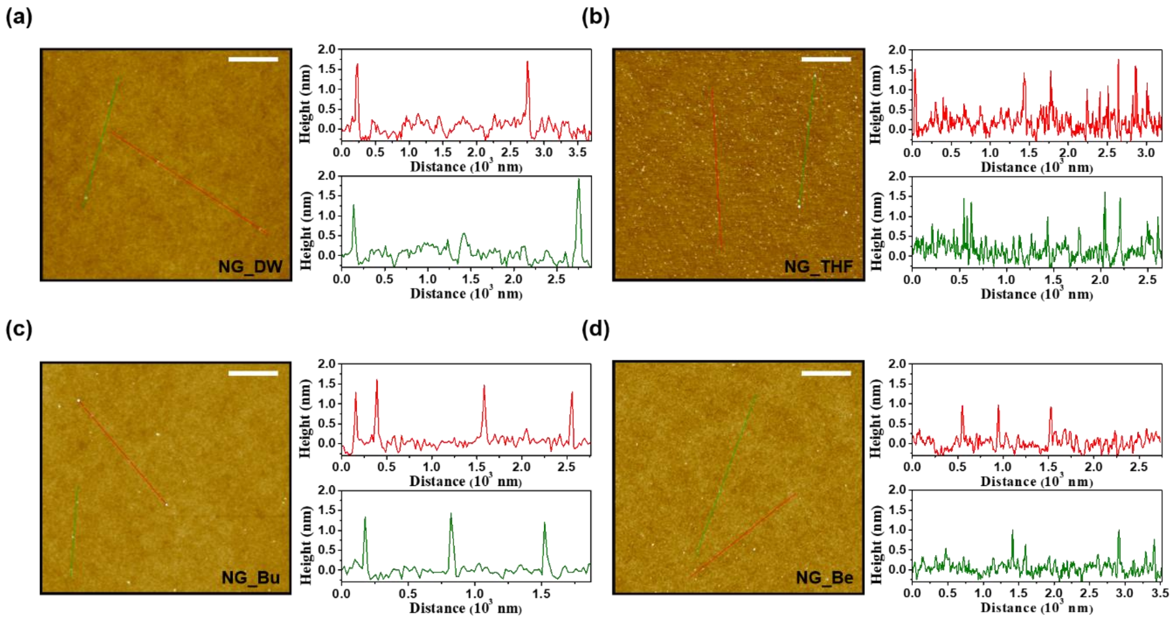

Supplementary Figure 20 AFM height images showing the corresponding height profiles of **(a)** NG_DW, **(b)** NG_THF, **(c)** NG_Bu and **(d)** NG_Be (scale bar: 1 μm).

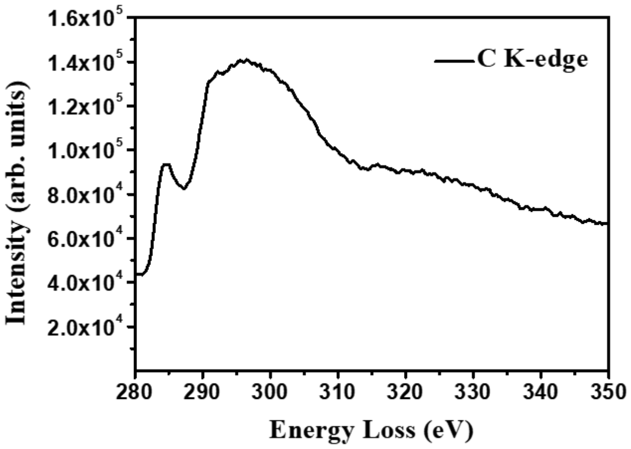

Supplementary Figure 21 C *K*-edge EELS spectrum of NG_Be. The C *K*-edge region exhibits one clear peak at approximately 284.8 eV, corresponding to the transition from the 1*s* to π^* state (1*s* → π^*), and another relatively broader peak at approximately 291.7 eV, attributed to the transition from the 1*s* to σ* state (1*s* → σ^*).[10]

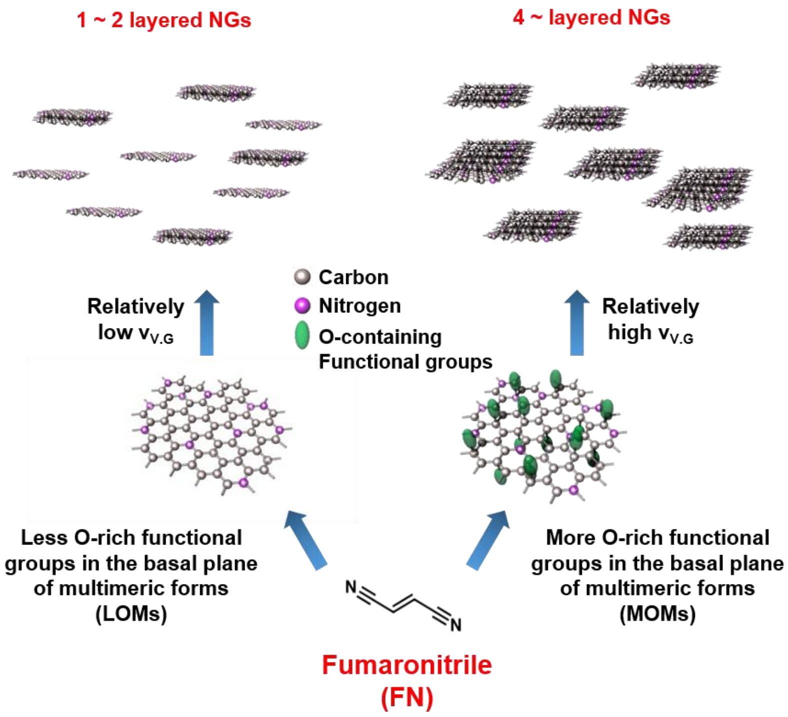

Supplementary Figure 22 Chemical configuration-dependent growth of NGs. Schematic illustration of two possible routes for the formation of NGs according to the chemical and structural configurations of their intermediate products.

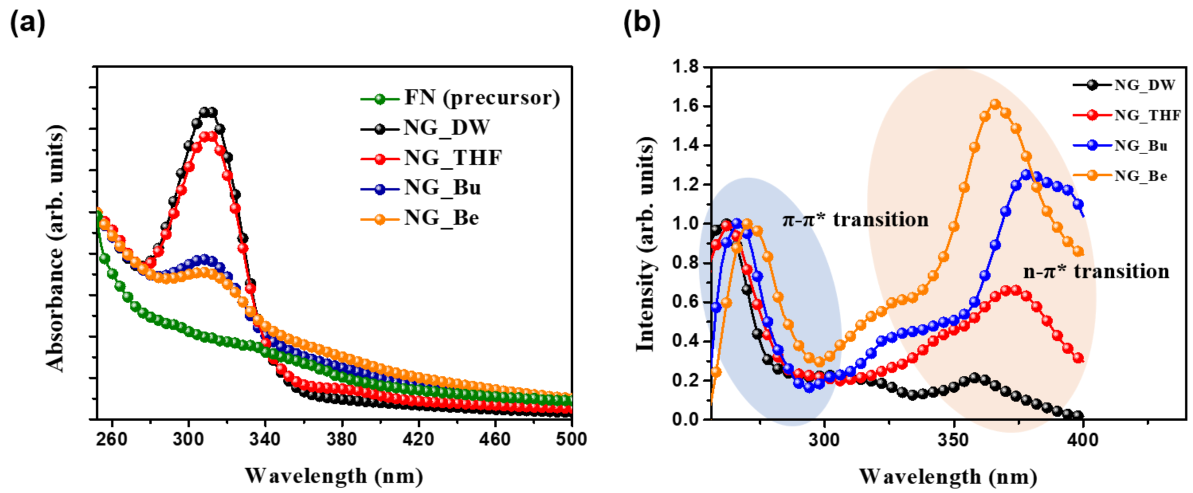

Supplementary Figure 23 (a) UV-vis absorption and **(b)** PLE spectra of NGs.

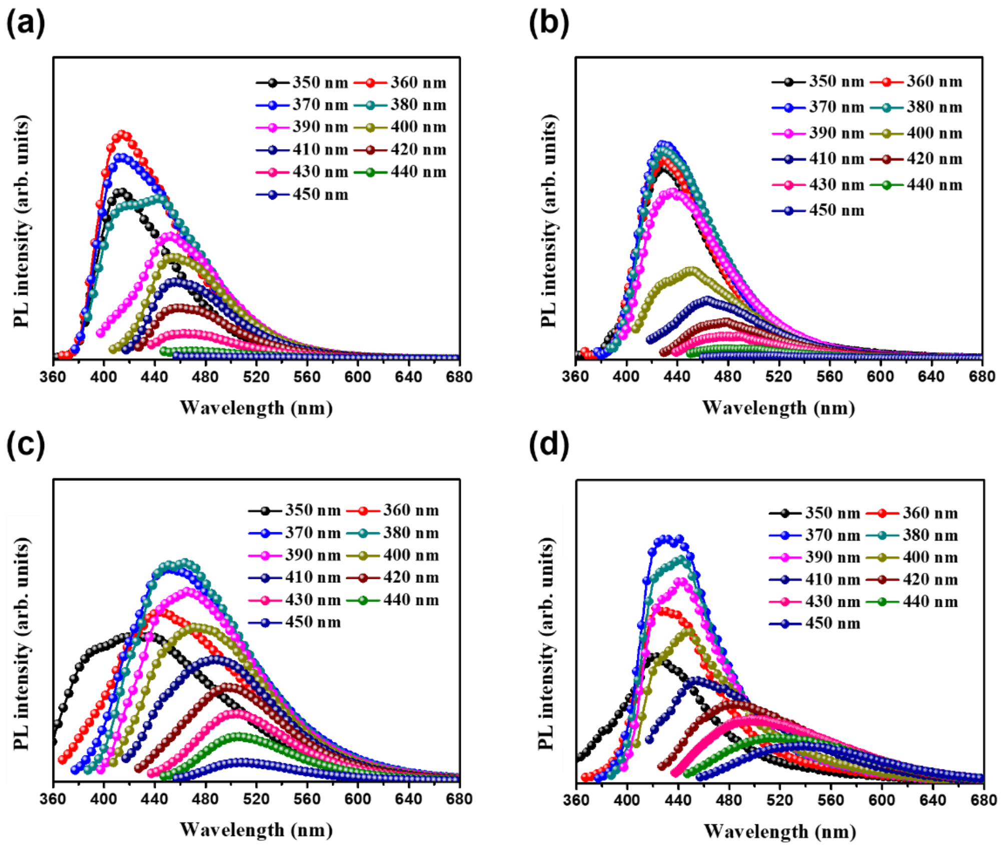

Supplementary Figure 24 PL spectra of NGs (**(a)** NG_DW, **(b)** NG_THF, **(c)** NG_Bu, and **(d)** NG_Be) at different excitation wavelengths.

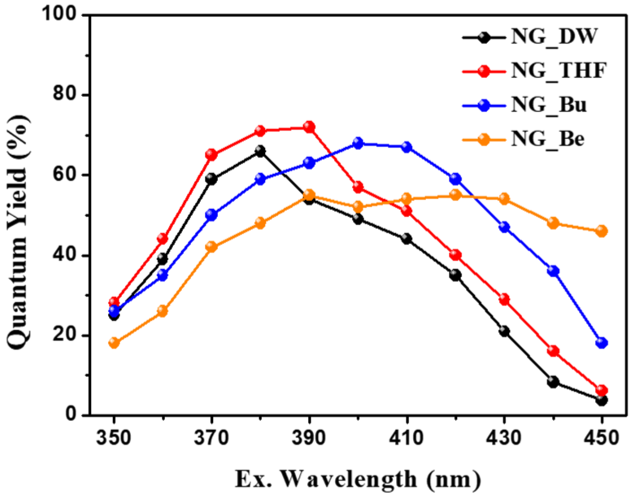

Supplementary Figure 25. Comparison of QYs of NGs as a function of λ_{ex}.

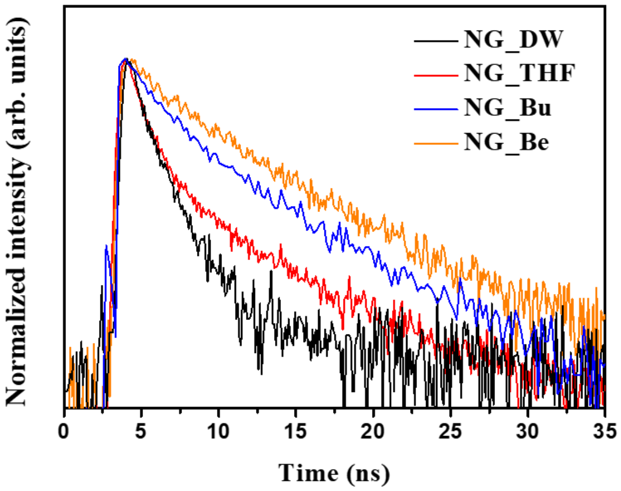

Supplementary Figure 26. TRPL decay curves of NGs monitored at 500 nm (λ_{ex} = 350 nm) and fitted with a tri-exponential function, indicating the presence of three PL decay sites with different probabilities of non-radiative decay. The average PL decay time ($\tau_{av,NGs}$) for each NG was calculated using the formula τ_{av} = $(A_1\tau_1^2+A_2\tau_2^2+A_3\tau_3^2)/(A_1\tau_1+A_2\tau_2+A_3\tau_3)$, where τ_1 (fast relaxation time), τ_2 (mid-relaxation time), and τ_3 (slow reaction time) are lifetimes (Supplementary Table 2).

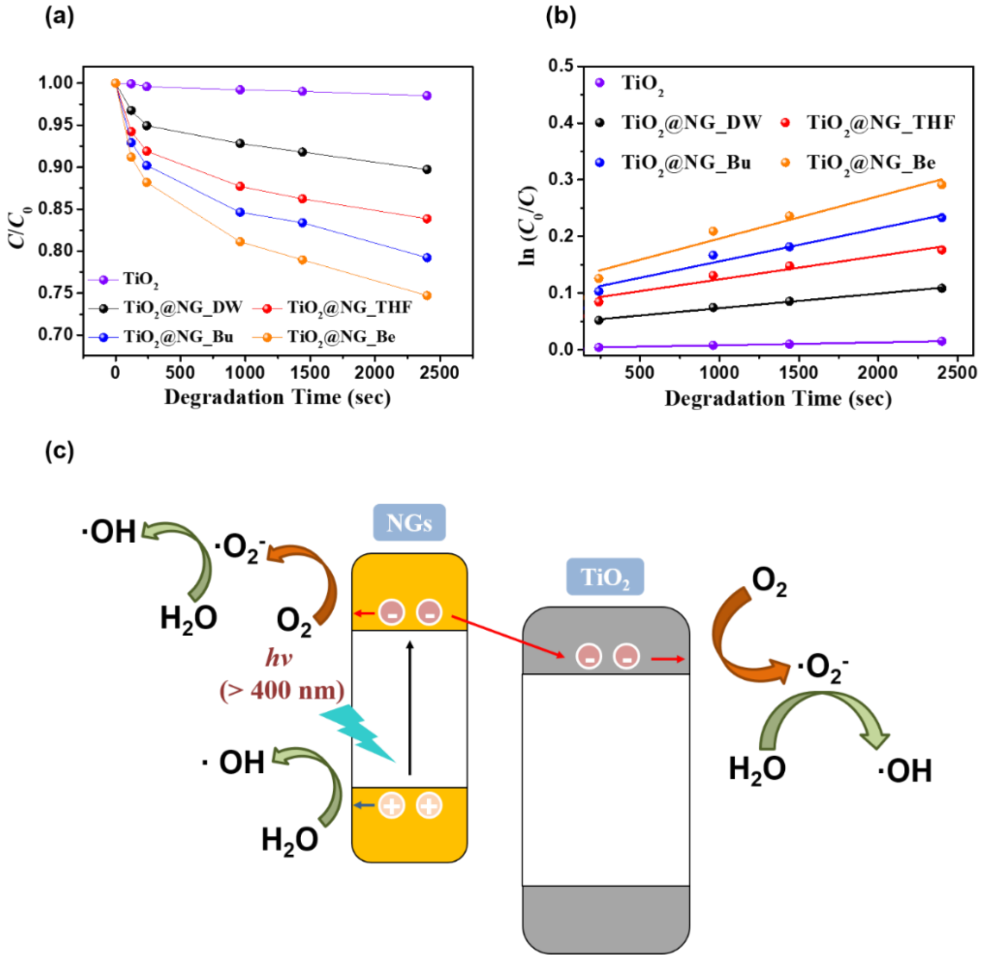

Supplementary Figure 27. (a) Comparison of photocatalytic degradations of RhB in the presence of different TiO_2-based photocatalysts under visible light irradiation (λ > 400 nm) and **(b)** their logarithmic notation for representing first-order kinetics. **(c)** Proposed pathway for the TiO_2@NGs-assisted photodegradation of organic pollutants under visible light irradiation.

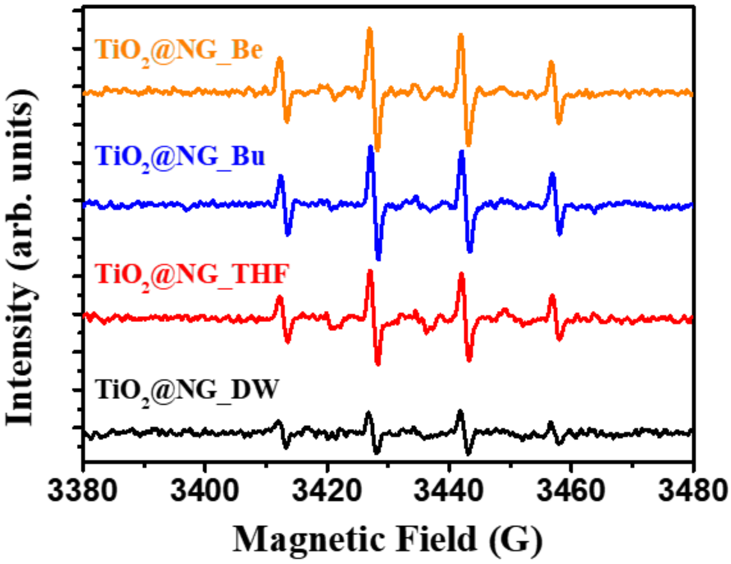

Supplementary Figure 28 EPR spectra of the TiO_2@NGs in aqueous dispersion for DMPO-•OH^- under visible light irradiation.

Supplementary Tables

Supplementary Table 1 Excitation energies (*E*), oscillator strengths (*f*), and transition coefficients (the percentage of the contributions of the two most important electronic transitions) of the first 5 lowest excited states of pristine and edge-functionalized C_7-GQDs.

	Pristine C_7			C_7-6OH			C_7-6NH_2		
	E (eV)	*f* (a.u.)	Contributions (%)	*E* (eV)	*f* (a.u.)	Contributions (%)	*E* (eV)	*f* (a.u.)	Contributions (%)
**S0 → S1**	-	0	-	-	0	-	3.239	0.0001	H → L (33.9)<br>H-1 → L+1 (39.1)
**S0 → S2**	-	0	-	-	0	-	-	0	-
**S0 → S3**	4.653	1.0412	H-1 → L+1 (35.8)<br>H → L (35.8)	4.363	1.0246	H-1 → L+1 (46.5)<br>H → L (46.2)	4.117	1.0729	H → L+1 (48.4)<br>H-1 → L (48.3)
**S0 → S4**	4.653	1.0412	H-1 → L (35.8)<br>H → L + 1 (35.8)	4.366	1.0375	H-1 → L (46.6)<br>H → L + 1 (46.6)	4.117	1.0730	H → L+2 (48.4)<br>H-1 → L+1 (48.3)
**S0 → S5**	-	0	-	4.562	0.0004	H-1 → L+2 (25.0)<br>H → L+3 (24.5)	-	0	-
	C_7-6COOH			C_7-6CONH_2			C_7-6LCOOH		
**S0 → S1**	3.365	0.0008	H → L (32.7)<br>H-1 → L+1 (28.3)	3.231	0.0332	H → L (60.8)<br>H-1 → L+1 (18.9)	3.139	0.0016	H → L (44.2)<br>H-1 → L+1 (39.1)
**S0 → S2**	3.612	0.0028	H-1 → L (32.8)<br>H → L+1 (29.0)	3.360	0.0539	H → L+1 (57.9)<br>H-1 → L (24.9)	3.296	0.0001	H-1 → L (43.8)<br>H → L+1 (41.7)
**S0 → S3**	4.304	1.1385	H → L+1 (36.4)<br>H-1 → L (33.3)	3.713	0.4413	H-1 → L+1 (44.5)<br>H → L (11.5)	3.787	1.7040	H-1 → L+1 (45.5)<br>H → L (39.3)
**S0 → S4**	4.312	1.1126	H-1 → L+1 (38.1)<br>H → L (32.9)	3.840	0.3043	H-1 → L (51.7)<br>H → L + 1 (14.7)	3.824	1.8992	H → L+1 (44.0)<br>H-1 → L (42.6)
**S0 → S5**	4.534	0.0487	H-2 → L (32.3)<br>H → L+2 (19.2)	4.072	0.0518	H-2 → L (45.2)<br>H-3 → L + 1 (20.6)	4.021	0.0779	H → L+2 (51.8)<br>H -1 → L+3 (10.0)
	C_7-6LCONH_2			C_7-6LCN					
**S0 → S1**	3.187	0.0017	H → L (48.0)<br>H-1 → L+1 (42.3)	3.152	0.0019	H → L (42.8)<br>H-1 → L+1 (37.1)			
**S0 → S2**	3.367	0.0015	H-1 → L (46.2)<br>H → L+1 (46.2)	3.318	0.0010	H-1 → L (42.4)<br>H → L+1 (39.4)			
**S0 → S3**	3.885	1.7043	H-2 → L (46.7)<br>H → L (40.9)	3.825	1.7494	H-1 → L+1 (47.9)<br>H → L (40.8)			
**S0 → S4**	3.921	1.8733	H-1 → L (44.7)<br>H → L+1 (44.5)	3.854	1.9361	H → L+1 (46.2)<br>H-1 → L (43.6)			
**S0 → S5**	4.166	0.0634	H → L+2 (42.2)<br>H-2 → L+1 (14.0)	4.074	0.0775	H → L+2 (34.0)<br>H -1 → L+2 (22.7)			

Supplementary Table 2 PL lifetimes of NGs dissolved in ethanol.

Sample	τ_1 (ns)	a_1	τ_2 (ns)	a_2	τ_3 (ns)	a_3	τ_{avg} (ns)
**NG_DW**	1.232	0.217	6.507	0.041	10.021	0.004	**4.3**
**NG_THF**	1.239	0.182	6.724	0.028	10.052	0.005	**4.5**
**NG_Bu**	1.253	0.042	6.687	0.119	10.286	0.017	**7.0**
**NG_Be**	1.203	0.234	5.295	0.039	26.668	0.009	**10.8**

Supplementary Discussion

Supplementary Discussion 1 Synthesis mechanism of NGs. In one reaction route (Multimerization 1), intermediate product 1 (Int-1), a kind of vinyl oligomer, is prepared via an addition multimerization reaction of FN (i.e., Multi-1: 1,4-Michael-type addition,[1,11,12] Multi-2: 1,2-nucleophilic addition on the nitrile group[13] and Multi-3: 1,4-addition through the nitrile group and the vinyl carbon[6]) when reactions are being performed in closed reaction vessels. During this reaction process, there is a drastic change in the Raman scattering profile corresponding to the C-H out-of-plane bending vibrations of FN (approximately 840 cm^{-1}) due to the depletion of the π-electrons (See Supplementary Figure 1), especially during 1,4-Michael-type addition multimerization.[13] On the other hand, in another reaction route (Multimerization 2), the transformation reaction of C≡N groups occurs via the addition of nucleophile molecule into electron-deficient C atoms under high temperature conditions to form amides, oximes, imidic acids, or even carboxylic acids (Int-2), which is determined from NMR data as shown in Supplementary Figure 2.[14,15] The resulting intermediates are not fully conjugated and do not possess graphitic structure, which are expected not to be fluorescent as shown in Supplementary Figure 3. In general, such an addition-elimination reaction occurs when the leaving group is attached to *sp* or sp^2 hybridized carbon center (nitrile and amide).

During these processes, most of the leading reactant of FN hydrolysis, i.e., H_2O, is assumed to be supplied from the background moisture of the air in reaction chamber, which later actively diffuses or permeates into the volume of reactant as the sealed chamber was pressurized by the high reaction temperature. Even in a rather dry atmosphere, the shortage can still be supplemented by the H_2O molecules generated during the condensation polymerization or cyclization of C≡N groups in intermediate oligomers via the dehydration process. In this regard, the effect of relative hymidity (26% ~ 69% RH) of air on the activation degree of FN was investigated by adding water vapor to a fixed concentration of FN (Figure S4). From this result, it was considered that, although the oxidation degree of FN can be slightly affected by the RH, the oxidation degree of FN can be more greatly affected by the type of solvent system. Consequently, due to the reliable supply of H_2O molecules by these mechanisms, further supplement of water into the synthesis, e.g., use of water as solvent, is found to be unnecessary. Also, based on previous reports[16,17] and our results (Figure S5), the effect of oxygen on the oxidation degree of C≡N group would be negligible.

Pre-NGs can then be produced by the carbonization or aromatization process of various oligomers, synthesized through amidation or aldol condensation reactions of various intermediate products, and via intramolecular dehydration or keto-enol tautomerisms.

Finally, after graphitization process at 170 °C (dehydrogenation, denitrogenation, and conversion to graphitic structures), four different coloured NG solutions could be obtained, as shown in Scheme 1 (NG_DW (solvent: DI-water), NG_THF (solvent: THF), NG_Bu (solvent: butylaldehyde), and NG_Be (solvent: benzaldehyde)).

Supplementary Discussion 2 Chemical configuration-dependent growth of NGs. It is noteworthy that there are significant thickness variations among the different NGs (Figure 2a). Especially, the average height of NG_Be is actually less than 1.0 nm (1~2 layers), suggesting that its vertical growth is effectively suppressed compared to other types of NGs. Until now, we have supposed that these features are likely to be related to the competitive reaction pathways for non-oxidative addition multimerization and solvent-assisted oxidation of FN. From the results about the chemical transformation of FN, we deduce the following simplified synthesis route of NGs in a closed-reaction system (Supplementary Figure 22).

Firstly, considering the composition analysis results of NGs, we can suppose that they are synthesized from little-oxidized multimeric monolayers (LOMs) and relatively more-oxidized multimeric forms (MOMs). Another important factor is that the preferential growth direction of NGs (lateral or vertical) is dependent on the variety of reaction kinetics, such as the 1) steric hindrance of functional groups in multimeric forms, 2) the molecular geometry of intermediate molecules, and 3) the reaction selectivity for the formation of electronically (or geometrically) stable graphitic nanostructures.[18,19]

Because the amide or oxime groups of nano-sized carbon clusters can be easily coupled to the O-containing groups attached on the basal or edge surface of different sheets, MOMs are more likely to contribute to the bidirectional growth of NGs than LOMs. That is to say, the formation of stitched graphitic sheets are determined by the reaction selectivity during the multimerization and carbonization of FN. Actually, the average height of NG_Be samples (Figure 2a), which contain the least oxygen content among the NGs, is actually less than 1.0 nm (1~2 layers), indicating that compared with other types of samples, vertical growth of NG_Be is effectively suppressed by a relatively small quantity of MOMs during the formation process.

Additionally, the excessive number of O-containing groups generated from oxidation reaction of FN are prone to suppressing the intermolecular cross-linking reactions during the carbonization process.[20,21] In other words, the number of oxygen-free oligomers has a strong influence on the formation of graphitic carbon structures without any structural (point, line, and surface) defects in NGs. From the result of XPS and NEXAFS analyses (Figure 2c and Supplementary Figure 7), drastic changes in molecular configurations of NGs are observed, such as the sp^2 C-C bond/sp^3 C-C bond ratio, and the relative intensities of σ* resonances (σ*/π*). Although the obtained fractions are not absolute values, we can at least deduce that the O-containing groups of intermediate carbon structures can play an important role as an inhibitor of the formation of graphitic structures without structural defects, which may lead to the degradation (or modulation) of photophysical or photochemical properties in NGs. Further study on the mechanism is ongoing in our laboratories.

Supplementary Discussion 3 Emission features of NGs. The calculated quantum yields (QYs) as a function of λ_{ex} (350 ~ 450 nm) are presented in Supplementary Figure 25. The values of each NGs are similar to or even higher than those of reported carbon-based QDs.[22-26] One of the plausible reasons is that the radiative recombination from GQDs is substantially attributed to electronic transitions between the sp^2 clusters and the boundaries of heteroatomized regions.[27] In other words, their small isolated sp^2 clusters act as interfacial junctions between the p- and n-type domains for recombination in the QDs. Therefore, on the basis of underlying physics, the relatively high QYs of our NGs under visible-light irradiation can be assumed to be associated with the relatively larger number of isolated nano-sized junctions between the p- and n-type, introduced by the edge functional groups of NGs as well as substitutional N atoms in their basal plane.

Supplementary Discussion 4 Photocatalytic activity of TiO_2-based photocatalysts. Owing to the higher LUMO level above the bottom of conduction band (CB) in TiO_2,[28] providing an energy difference of 0.5 eV or larger for an effective electron transfer from the CB of the NGs (donor) to the CB of TiO_2 (acceptor), the acceptor can promote 1) exciton dissociation and 2) charge-separation processes, and it can also draw free electrons at the donor-acceptor interface, as shown in Supplementary Figure 27c. After photogenerated electrons are successfully transferred to the neighbouring TiO_2 NPs, electrons are trapped by adsorbed O_2 molecules on

the TiO_2 surface to form superoxide anion radicals ($\bullet O_2^-$), which can be converted to •OH radicals, while the holes left in NGs form •OH radicals from H_2O molecules.

Supplementary Notes

Supplementary Note 1 Dynamics of solvent relaxations. According to the Lippert equation, the inhomogeneous broadening of PL spectra depends on solvent dipole, temperature, and emitter dipole moment upon excitation, which can be expressed as follow,[29]

$$\Delta\bar{\upsilon} = \frac{(\mu_e - \mu_g)^2}{ca^3}\left(\frac{2}{h}\right)\left[\frac{\varepsilon-1}{2\varepsilon+1} - \frac{n^2-1}{2n^2+1}\right] + constant \qquad (1)$$

with variables of $\Delta\mu$ (μ_e-μ_g, change in the dipole moment of the emitter upon excitation), *c* (speed of light), *a* (Onsager sphere radius), *h* (Planck's constant), and ε (dielectric constant of the solvent).

Supplementary Note 2 Poisson's equation. The electrical potential (V_{BB}) of a point (*x*,*y*,*z*) in the space charge region can be expressed by Poisson's equation,[30]

$$\frac{\partial^2 V_{BB}(x,y,z)}{\partial x^2} + \frac{\partial^2 V_{BB}(x,y,z)}{\partial y^2} + \frac{\partial^2 V_{BB}(x,y,z)}{\partial z^2} = -\frac{\rho}{\varepsilon_r\varepsilon_0} \qquad (2)$$

where ρ, ε_r, and ε_0 are the space charge density, relative dielectric constant, and vacuum permittivity, respectively. For simplification, 1D space charge distributions are often considered as follows,

$$V_{BB}(x) \propto -\frac{\rho}{2\varepsilon_r\varepsilon_0}x^2 \qquad (3)$$

where V_{BB} is a direct function of *x*-coordinates and also influenced by ρ, which is associated with surface atomic structures and compositions.

Supplementary References

1. Silva, C. *et al.* Characterization of the products of aniline peroxydisulfate oligo/polymerization in media with different pH by resonance Raman spectroscopy at 413.1 and 1064 nm excitation wavelengths. *J. Raman Spectrosc.* **42**, 1653-1659 (2011).
2. Mills, C. A. *et al*. The preparation and characterisation of polymeric macrostructures (command surfaces) using electropolymerisation. *J. Mater. Chem.* **10**, 1551-1554 (2000).
3. Li, Y. *et al.* Nitrogen-doped graphene quantum dots with oxygen-rich functional groups. *J. Am. Chem. Soc.* **134**, 15-18 (2012).
4. Favaro, M. *et al.* TiO_2/graphene nanocomposites from the direct reduction of graphene oxide by metal evaporation. *Carbon* **68**, 319-329 (2014).
5. Xue, Y. *et al*. Correlative study of critical reactions in polyacrylonitrile based carbon fiber precursors during thermal-oxidative stabilization. *Polym. Degrad. Stab.* **98**, 219-229 (2013).
6. Yildiz, E. *et al*. A soluble conducting polymer of 4-(2,5-di(thiophen-2-yl)-1*H*-pyrrol-1-yl)benzenamie and its multichromic copolymer with EDOT. *J. Electroanal. Chem.* **612**, 247-256 (2008).
7. Kang, H. C. *et al*. Enhanced electrical conductivity of polypyrrole prepared by chemical oxidative polymerization: effect of the preparation technique and polymer additive. *Polymer* **41**, 6931-6934 (2000).
8. Dalton, S. *et al*. Thermal stabilization of polyacrylonitirile fibres. *Polymer* **40**, 5531-5543 (1999).
9. Misra, N *et al*. Vibrational analysis of boldine hydrochloride using QM/MM approach. *Spectroscopy* **24**, 483-499 (2010).
10. Majumder, S *et al*. Magnetization enhancement of Fe_3O_4 by attaching onto graphene oxide: An interfacial effect. *J. Phys. Chem. C* **122**, 21356-21365 (2018).
11. Pardo, L. *et al*. Mechanisms of nucleophilic addition to activated double bonds: 1,2- and 1,4-Michael addition of ammonia. *J. Am. Chem. Soc.* **115**, 8263-8269 (1993).
12. Mills, C. A. *et al*. The preparation and characterisation of polymeric macrostructures (command surfaces) using electropolymerisation. *J. Mater. Chem.* **10**, 1551-1554 (2000).
13. Yang, Z. *et al*. Intrinsically soluble copolymers with well-defined alternating substituted p-phenylenevinylene and ethylene oxide blocks. *Macromolecules* **26**, 6570-6575 (1993).
14. Franchuk, I. F. A study of hydrogen bonds in melts of maleic acid and some acid salts. *J. Appl. Spectrosc.* **28**, 499-502 (1978).

15. Li, J. W. *et al.* Effect of ultraviolet irradiation on the characteristics and trihalomethanes formation potential of humic acid. *Water Res.* **30**, 347-350 (1996).

16. Zhang, W. *et al.* Sttudy of oxide-based catalysts for the oxidative transformation of acetonitrile to acrylonitrile with CH_4. *J. Catal.* **182**, 70-81 (1999).

17. Smirniotis, P. G. *et al*. Study of the oxidative methylation of acetonitrile to acrylonitrile with CH_4 over Li/MgO catalysts. *Appl. Catal. A: Gen.* **176**, 63-73 (1999).

18. Favaro, M. *et al*. Multiple doping of graphene oxide foams and quantum dots: new switchable systems for oxygen reduction and water remediation. *J. Mater. Chem. A* **3**, 14334-14347 (2015).

19. Frère, Y. *et al*. Reaction kinetics of polymer substitutents. Macromolecular steric hindrance effect in quaternization of poly(vinylpyridine). *Macromoleulces* **25**, 3184-3189 (1992).

20. Wang, J. *et al*. Effects of oxygen content in the atmosphere on thermal oxidative stabiliziation of polyacrylonitrile fibers. *RSC Adv.*, **6**, 73404-73411 (2016).

21. Bahl, O. P. *et al*. Characterization of oxidised pan fibres. *Carbon* **12**, 417-423 (1974).

22. Sun, Y-P. *et al.* Quantum-sized carbon dots for bright and colourful photoluminescence. *J. Am. Chem. Soc.* **128**, 7756-7757 (2006).

23. Briscoe, J. *et al*. Biomass-derived carbon quantum dot sensitizers for solid-state nanostructured solar cells. *Angew. Chem. Int. Ed.* **54**, 4463-4468 (2015).

24. Sun, H. *et al.* Highly photoluminescent amino-functionalized graphene quantum dots used for sensing copper ions. *Chem. Eur. J.* **19**, 13362-13368 (2013).

25. Li, Z. *et al*. A new fluorescent nitrogen-doped carbon dot system modified by the fluorophore-labeled ssDNA for the analysis of 6-mercaptopurine and Hg(II). *Biosensors and Bioelectronics* **74** 91-97 (2015).

26. Yeh, T-F. *et al*. Nitrogen-doped grapehen oxide quantum dots as photocatalysts for overall water-splitting under visible light illumination. *Adv. Mater.* **26**, 3297-3303 (2014).

27. Lim, E. C. *et al*. Role of $1 \rightarrow a_\pi$ transitions in spin-orbit coupling of aromatic amines: phosphorescence of aniline and its *N*-alkyl derivatives. *J. Phys. Chem. Phys.* **47**, 4726-4730 (1967).

28. Pan, D. *et al.* Efficient separation of electron-hole pairs in graphene quantum dots by TiO_2 heterojunctions for dye degradation. *ACS Sustainable Chem. Eng.* **3**, 2405-2413 (2015).

29. Mukherjee, S., Chattopadhyay, A., Samanta, A. & Soujanya, T. Dipole moment change of NBD group upon excitation studied using solvatochromic and quantum chemical approaches: Implications in membrane research. *J. Phys. Chem.* **98**, 2809-2812 (1994).

30. Zhang, Z. & Yates, J. T. Band bending in semiconductors: Chemical and physical consequences at surfaces and interfaces. *Chem. Rev.* **112**, 5520-5551 (2012).
